# Supplementary figures and images for: Glycogenesis and glyconeogenesis from glutamine, lactate and glycerol support human macrophage functions
Source: EMBO Rep. 2024 Oct 18;25(12):13. doi: 10.1038/s44319-024-00278-4 (PMC11624281; doi:10.1038/s44319-024-00278-4)

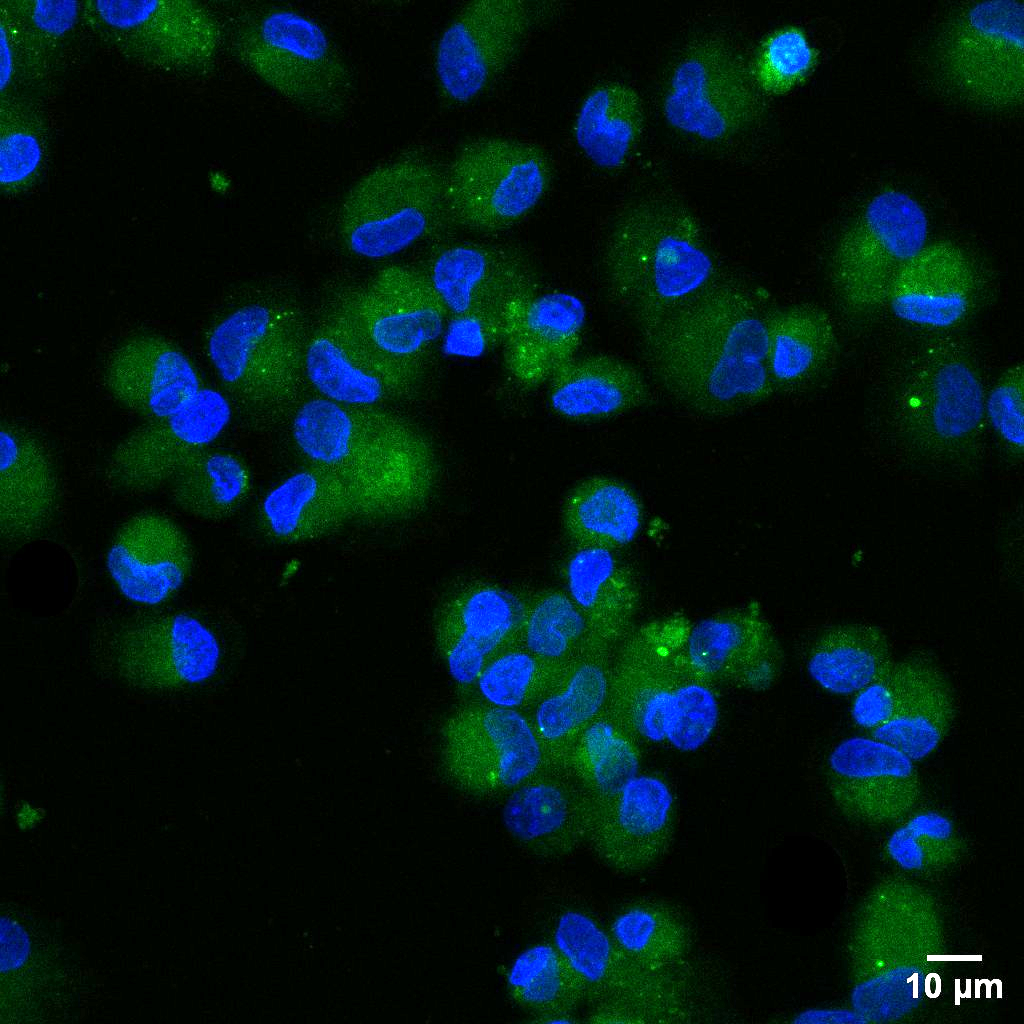

Supplement: Supplementary file 2 — Source data Fig. 1 [file 44319_2024_278_MOESM2_ESM.zip › Figure 1/1C/Confocal_M1 cells.jpg]

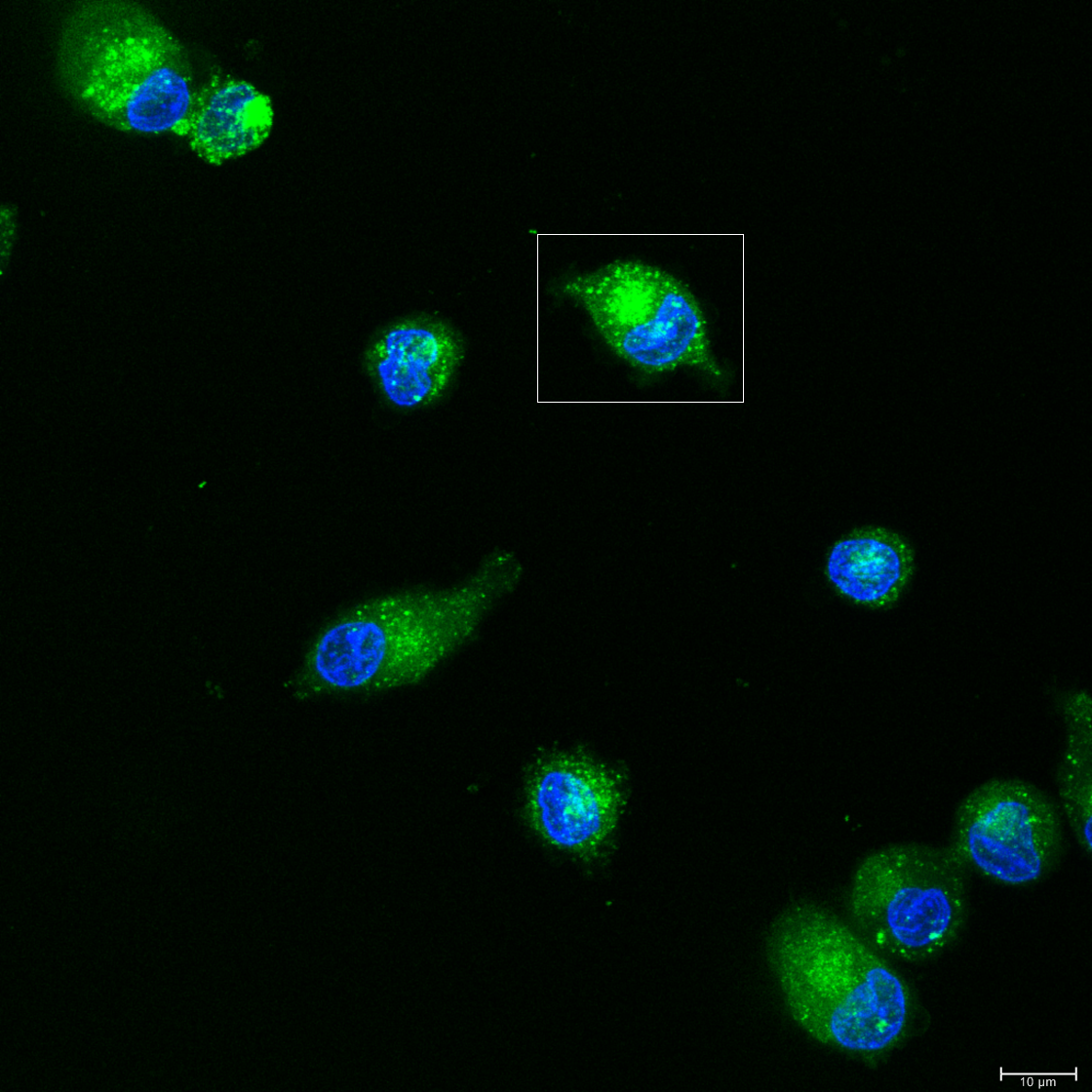

Supplement: Supplementary file 2 — Source data Fig. 1 [file 44319_2024_278_MOESM2_ESM.zip › Figure 1/1C/Confocal_M1 cells_zoomed.tif]

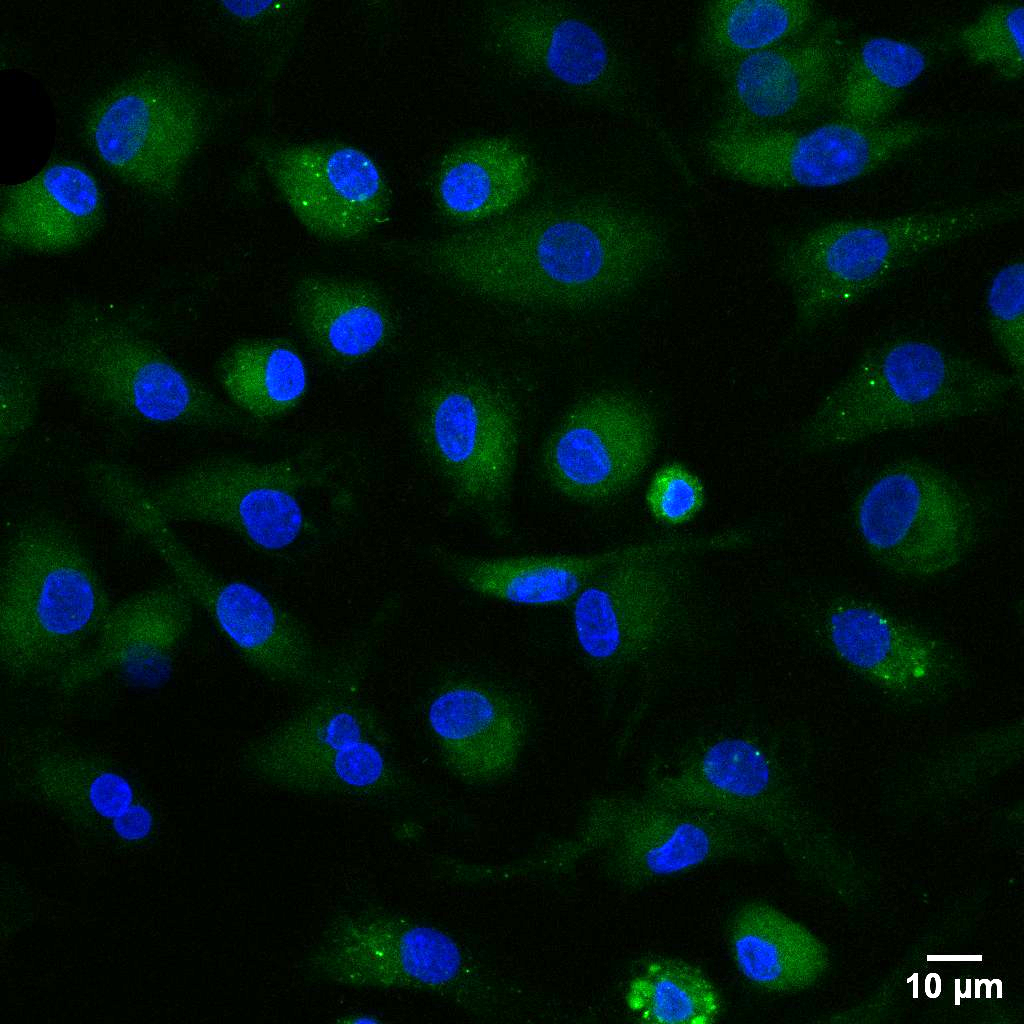

Supplement: Supplementary file 2 — Source data Fig. 1 [file 44319_2024_278_MOESM2_ESM.zip › Figure 1/1C/Confocal_M2 cells.tif]

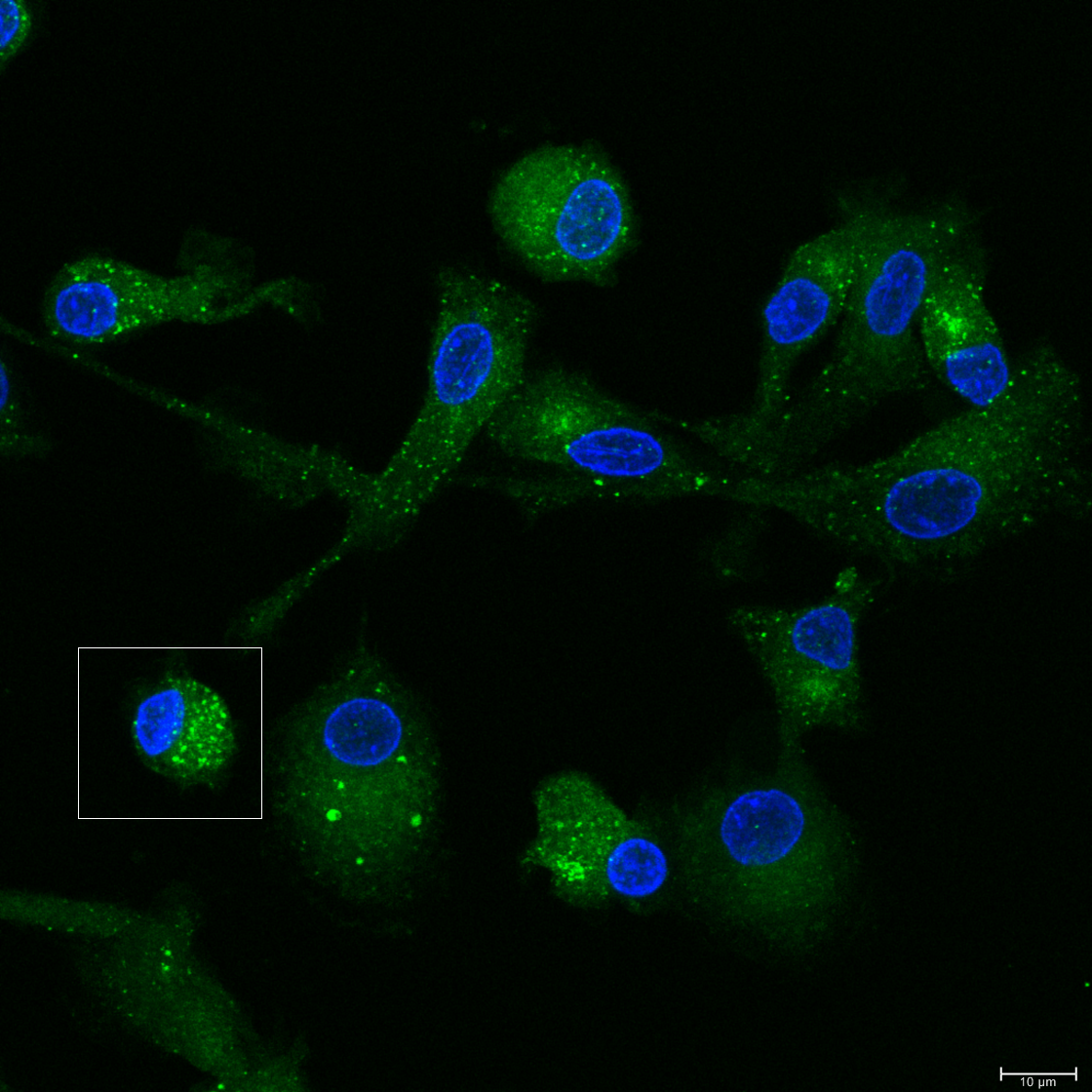

Supplement: Supplementary file 2 — Source data Fig. 1 [file 44319_2024_278_MOESM2_ESM.zip › Figure 1/1C/Confocal_M2 cells_zoomed.tif]

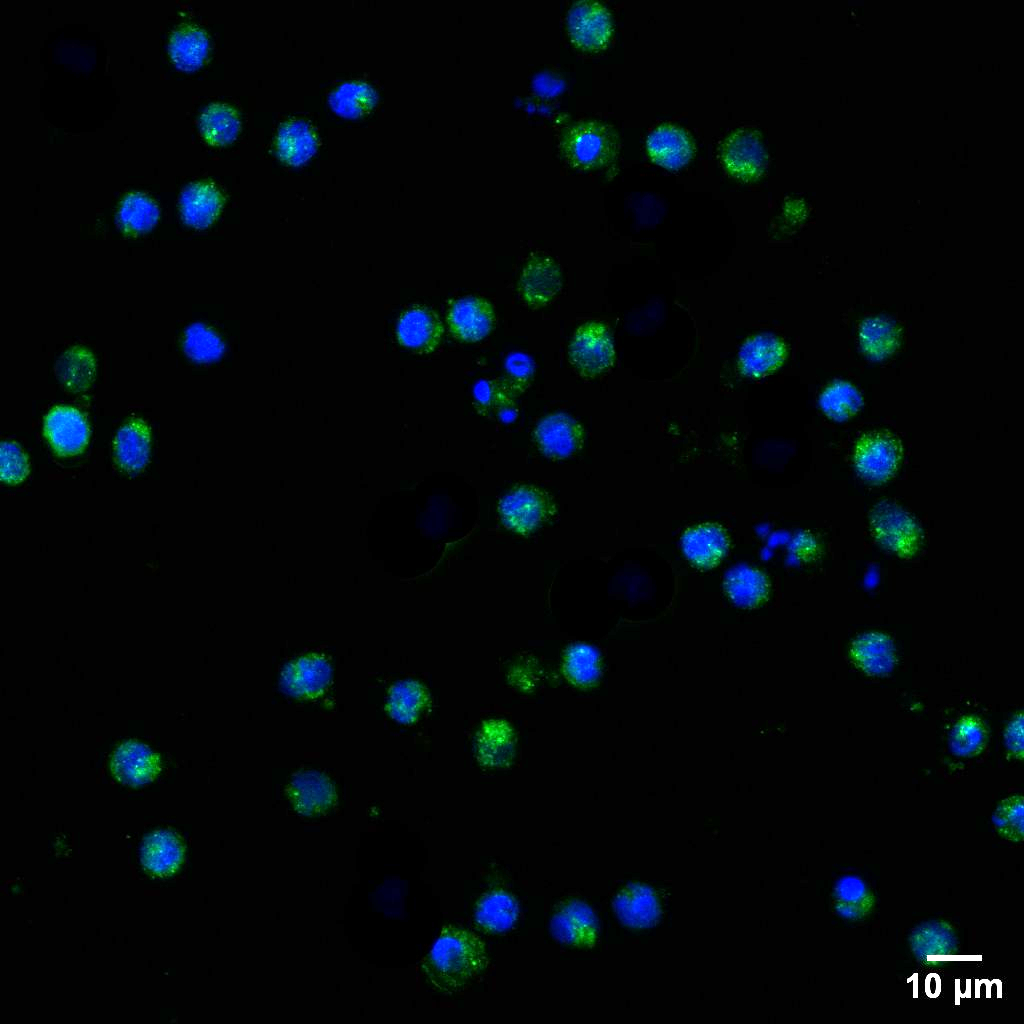

Supplement: Supplementary file 2 — Source data Fig. 1 [file 44319_2024_278_MOESM2_ESM.zip › Figure 1/1C/Confocal_Monocytes.tif]

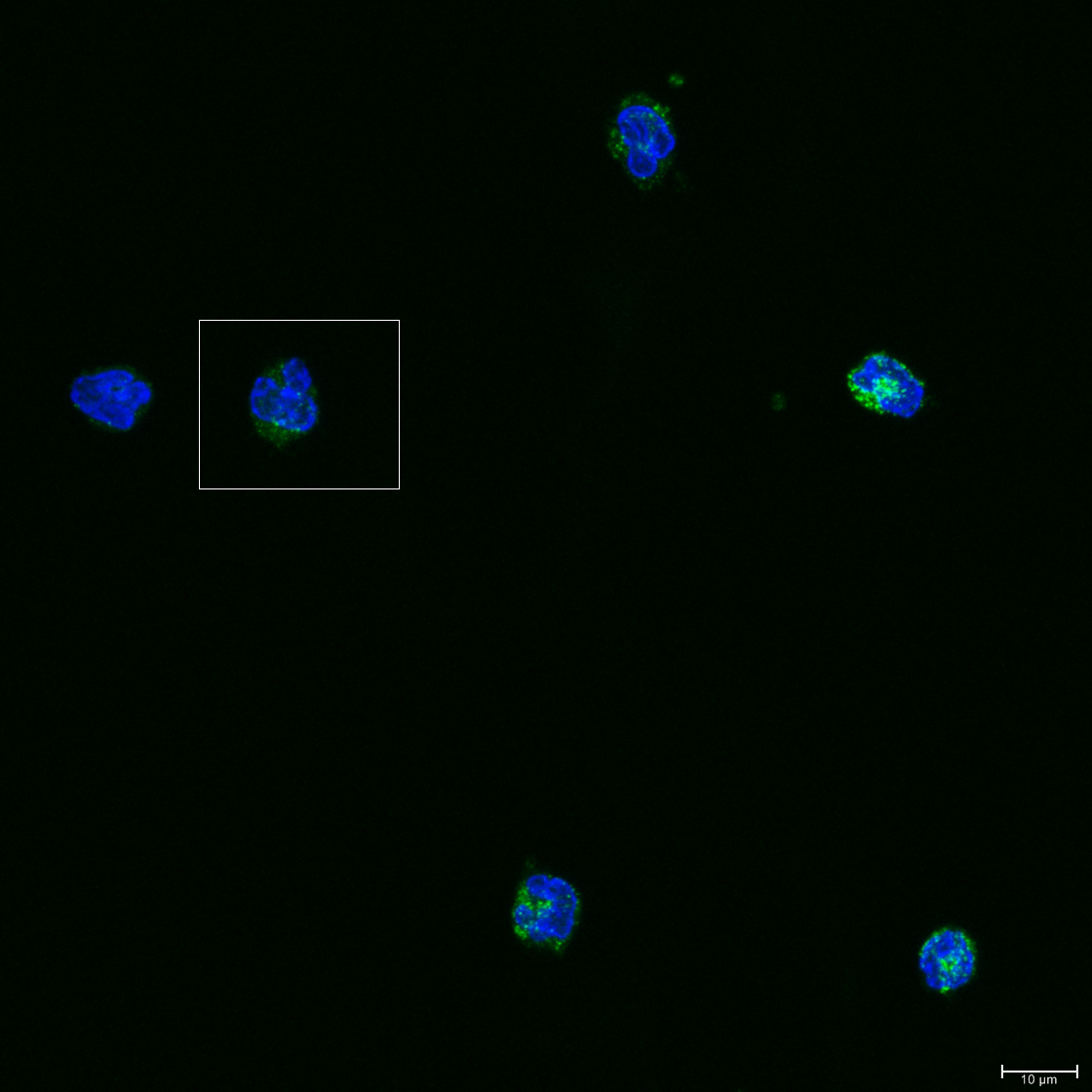

Supplement: Supplementary file 2 — Source data Fig. 1 [file 44319_2024_278_MOESM2_ESM.zip › Figure 1/1C/Confocal_Monocytes_zoomed.tif]

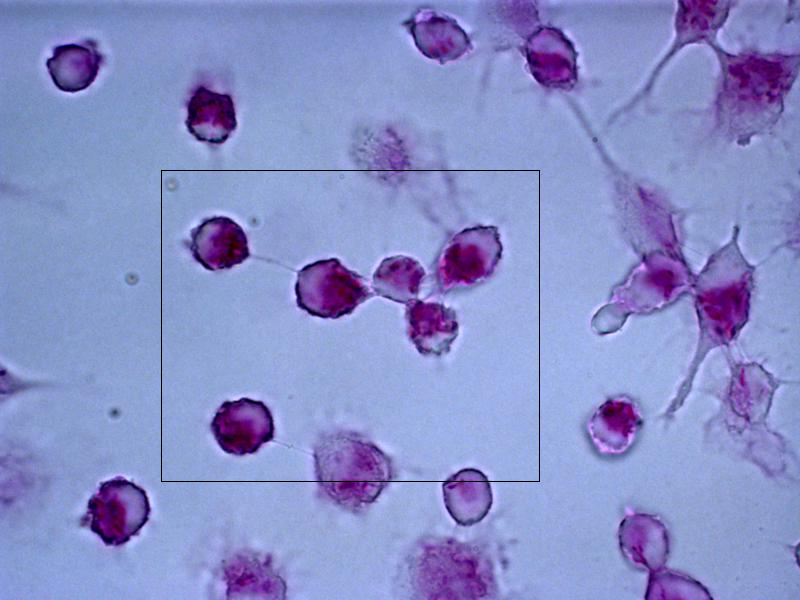

Supplement: Supplementary file 2 — Source data Fig. 1 [file 44319_2024_278_MOESM2_ESM.zip › Figure 1/1C/PAS_M1 cells.tif]

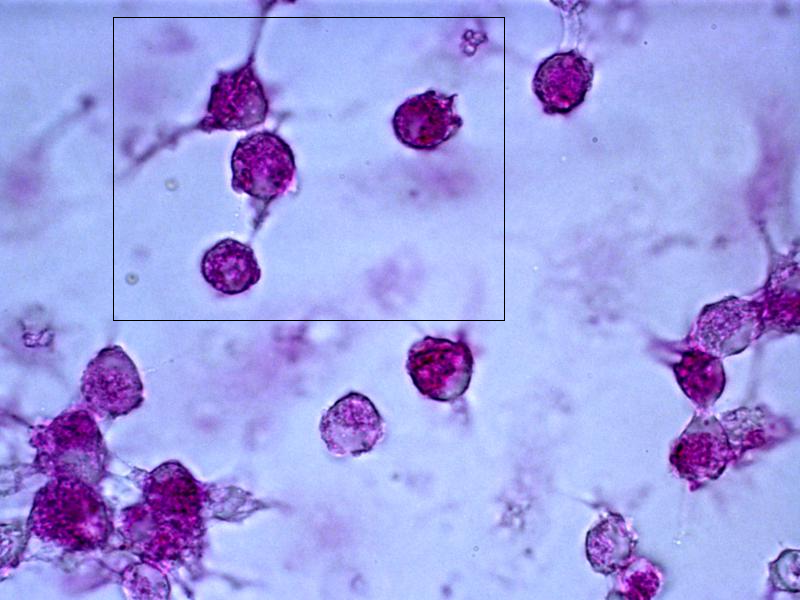

Supplement: Supplementary file 2 — Source data Fig. 1 [file 44319_2024_278_MOESM2_ESM.zip › Figure 1/1C/PAS_M2 cells.tif]

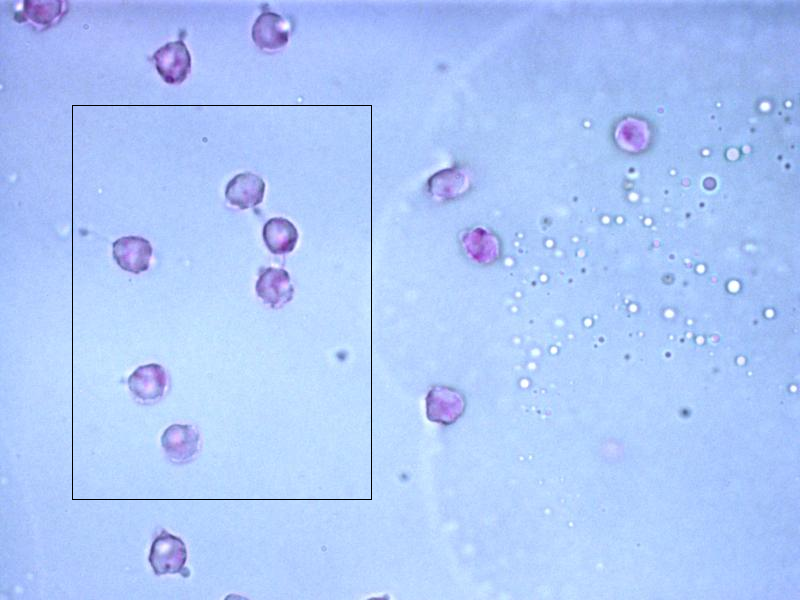

Supplement: Supplementary file 2 — Source data Fig. 1 [file 44319_2024_278_MOESM2_ESM.zip › Figure 1/1C/PAS_Monocytes.tif]

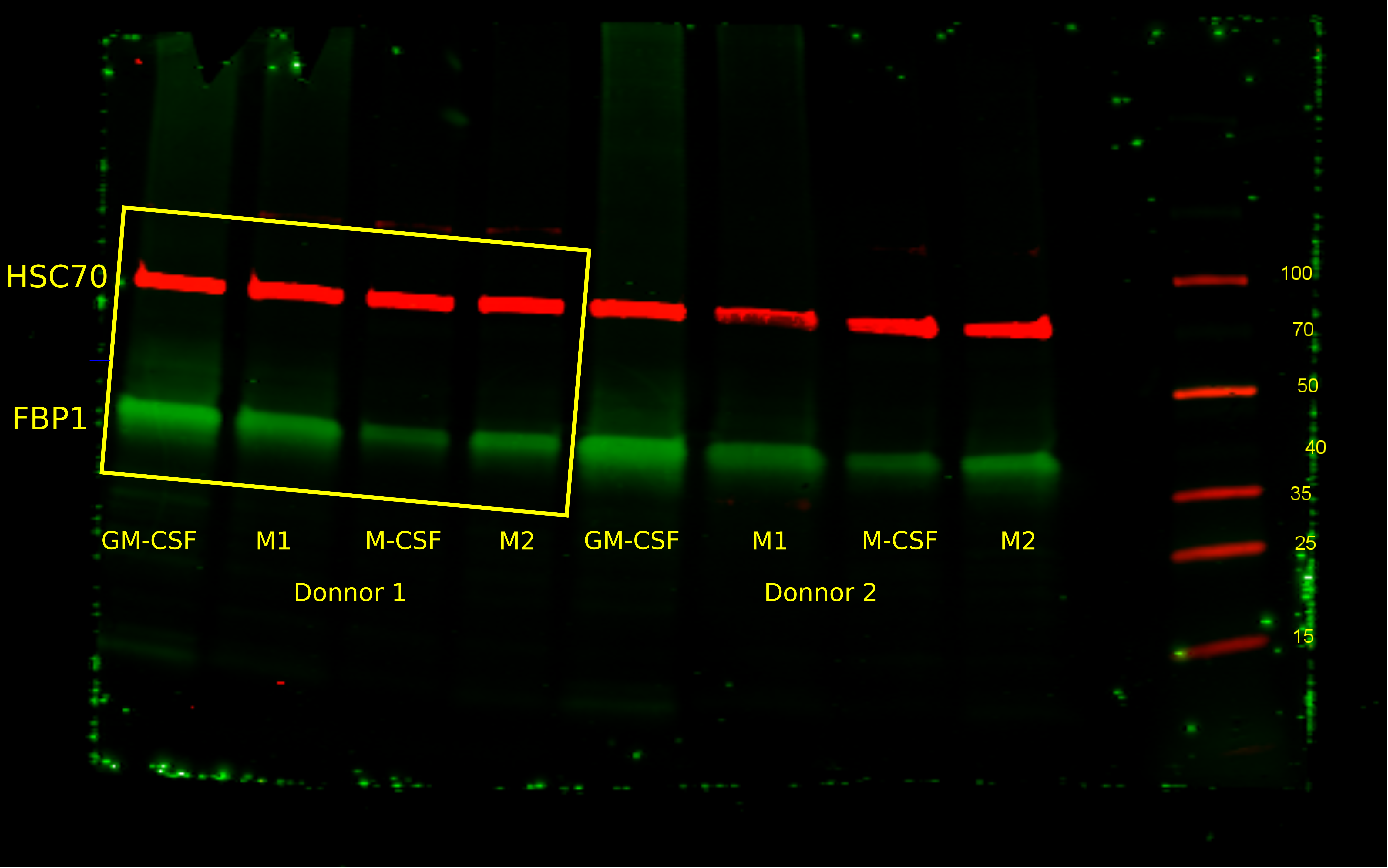

Supplement: Supplementary file 3 — Source data Fig. 2 [file 44319_2024_278_MOESM3_ESM.zip › Figure 2/2E/Western FBP1 HSC70.png]

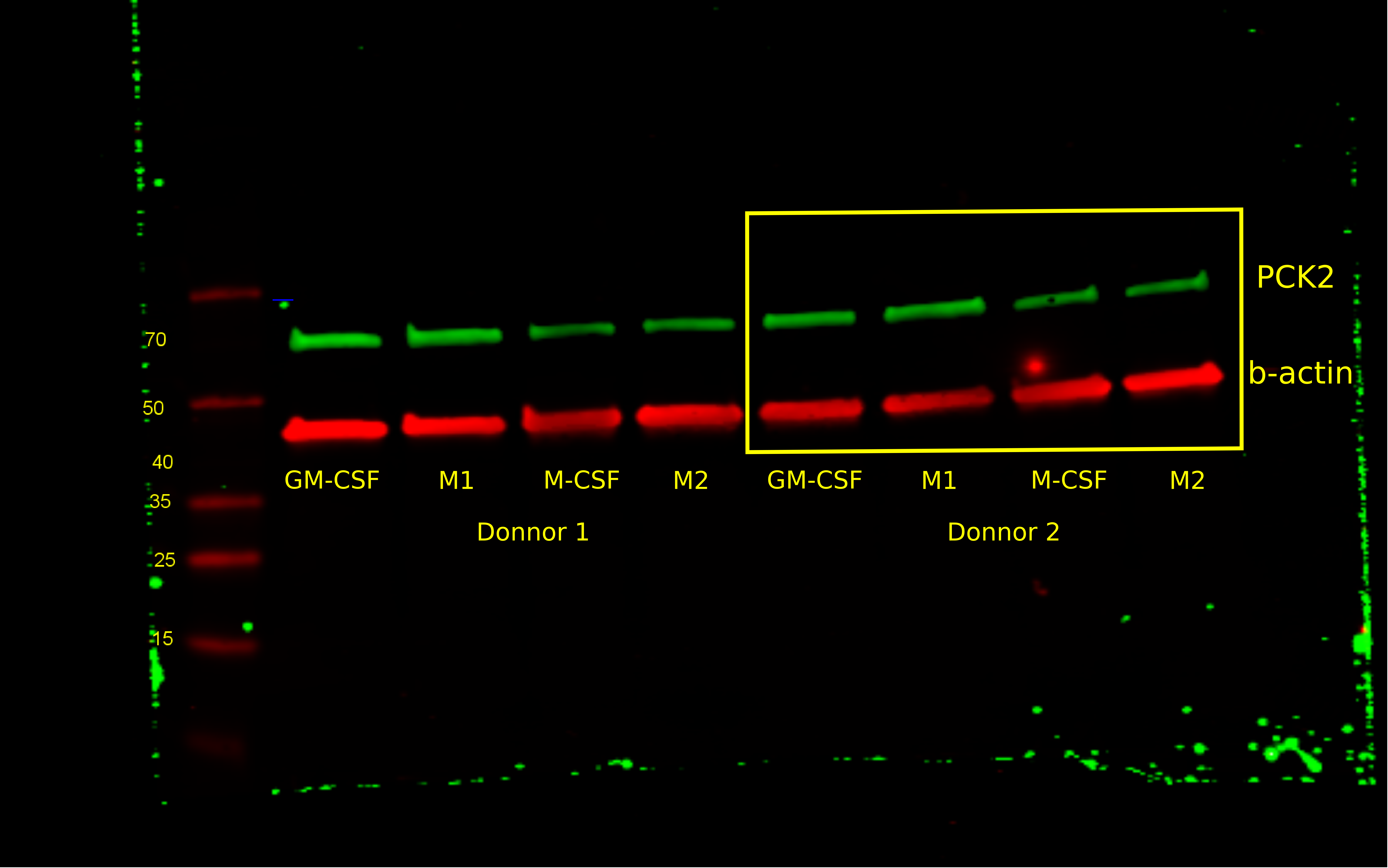

Supplement: Supplementary file 3 — Source data Fig. 2 [file 44319_2024_278_MOESM3_ESM.zip › Figure 2/2E/Western PCK2 b-actin.png]

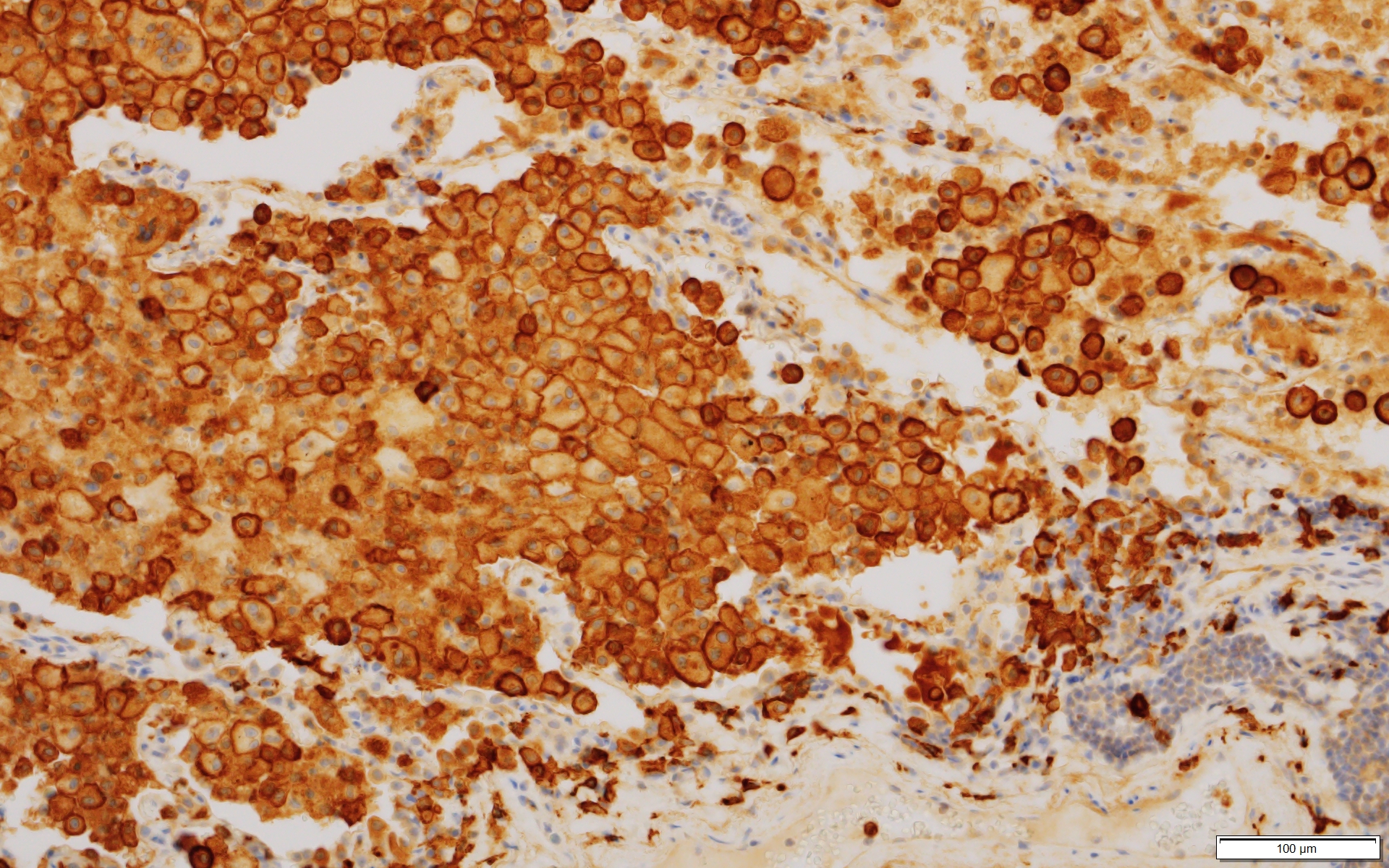

Supplement: Supplementary file 5 — Source data Fig. 4 [file 44319_2024_278_MOESM5_ESM.zip › Figure 4/4C/4A IHC CD163.jpg]

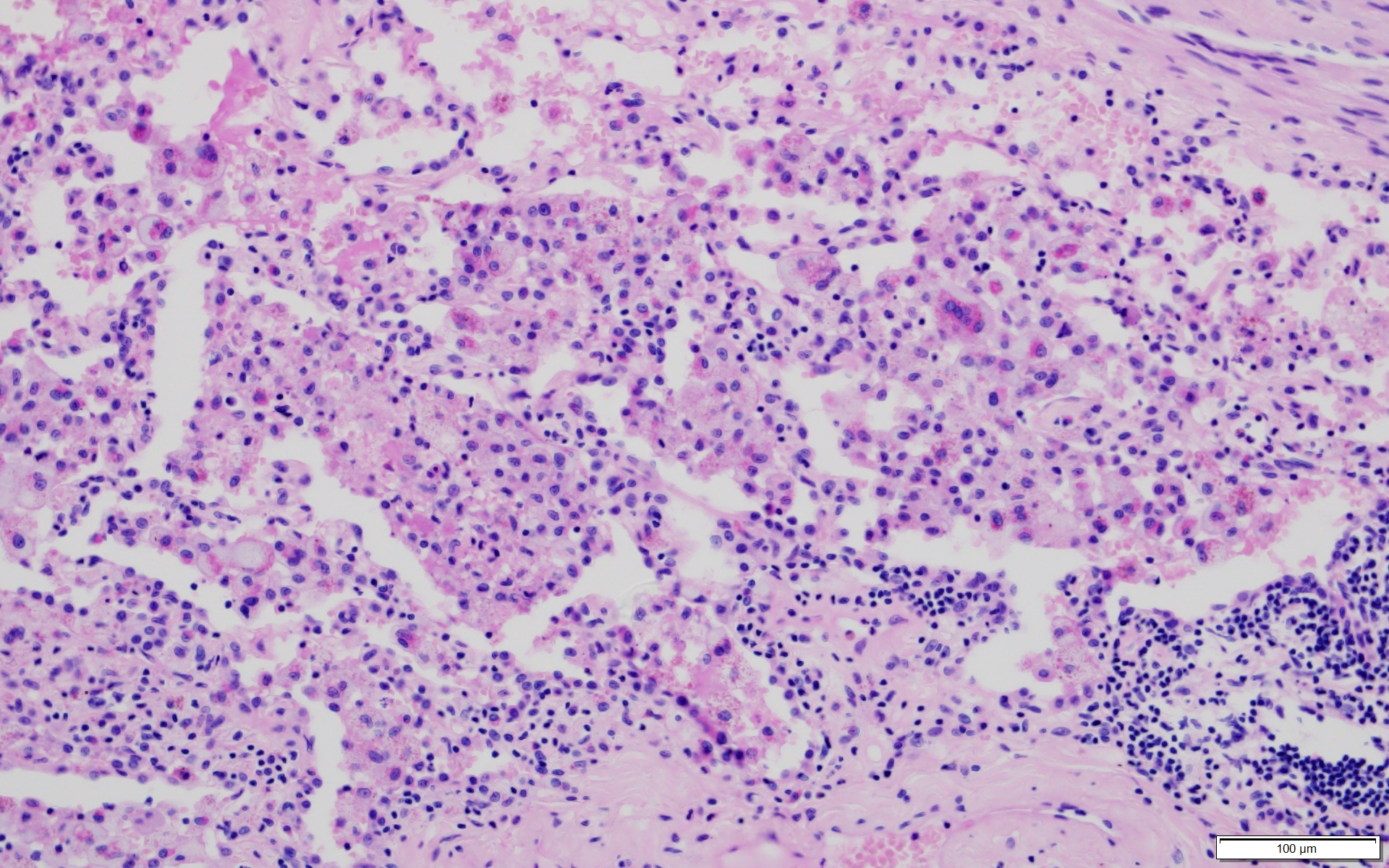

Supplement: Supplementary file 5 — Source data Fig. 4 [file 44319_2024_278_MOESM5_ESM.zip › Figure 4/4C/4A PAS Amylase.jpg]

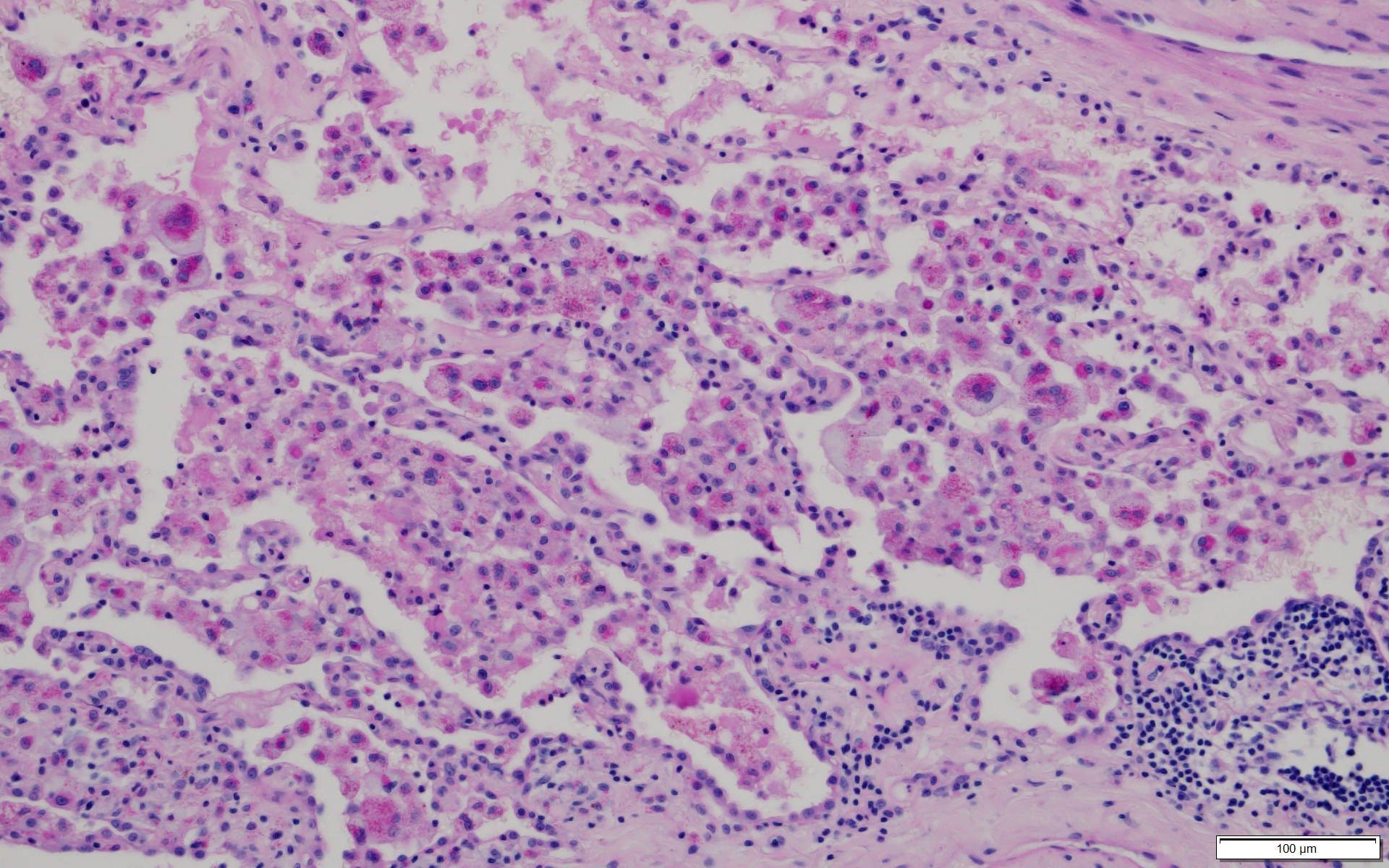

Supplement: Supplementary file 5 — Source data Fig. 4 [file 44319_2024_278_MOESM5_ESM.zip › Figure 4/4C/4A PAS.jpg]

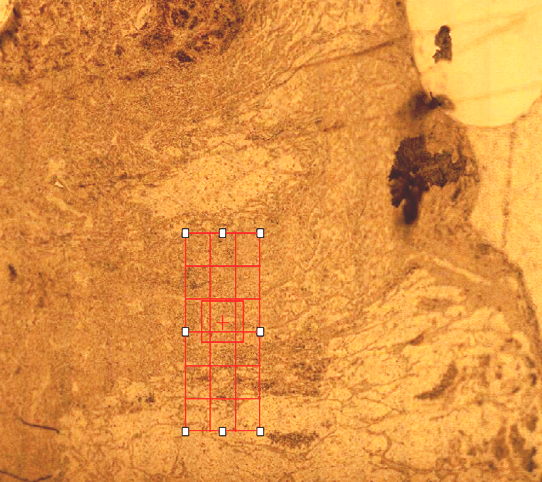

Supplement: Supplementary file 5 — Source data Fig. 4 [file 44319_2024_278_MOESM5_ESM.zip › Figure 4/4D/4E BrightField.tif]

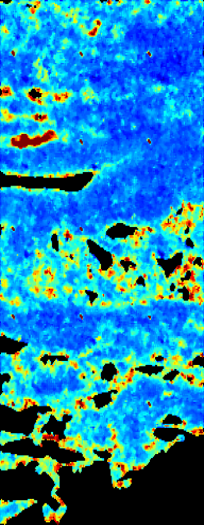

Supplement: Supplementary file 5 — Source data Fig. 4 [file 44319_2024_278_MOESM5_ESM.zip › Figure 4/4D/4E FTIR Glycogen.png]

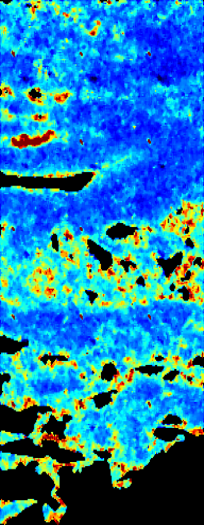

Supplement: Supplementary file 5 — Source data Fig. 4 [file 44319_2024_278_MOESM5_ESM.zip › Figure 4/4D/4E FTIR LacticAcid.png]

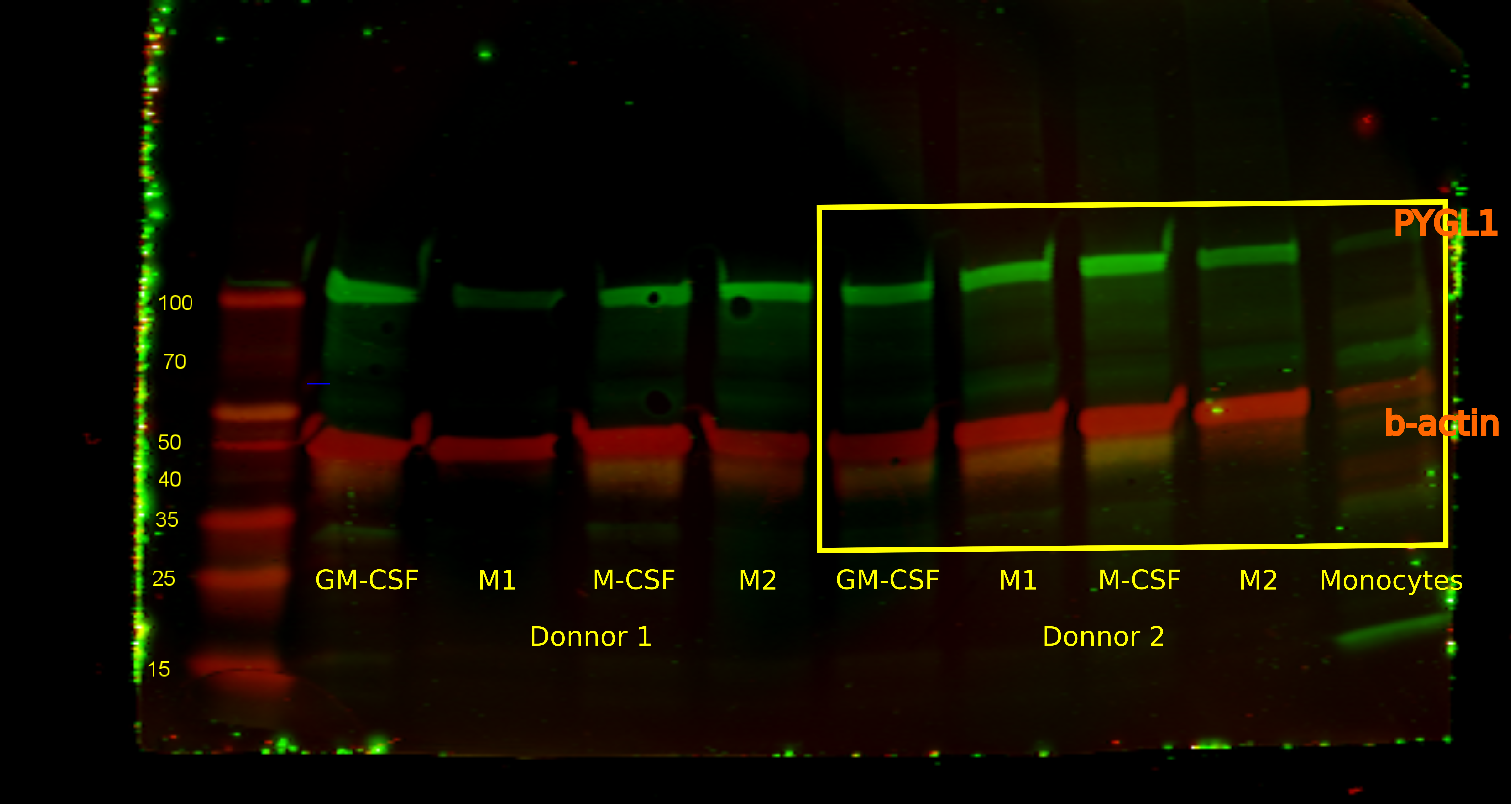

Supplement: Supplementary file 6 — Source data Fig. 5 [file 44319_2024_278_MOESM6_ESM.zip › Figure 5/5D/Western PYGL1 b-actin.png]

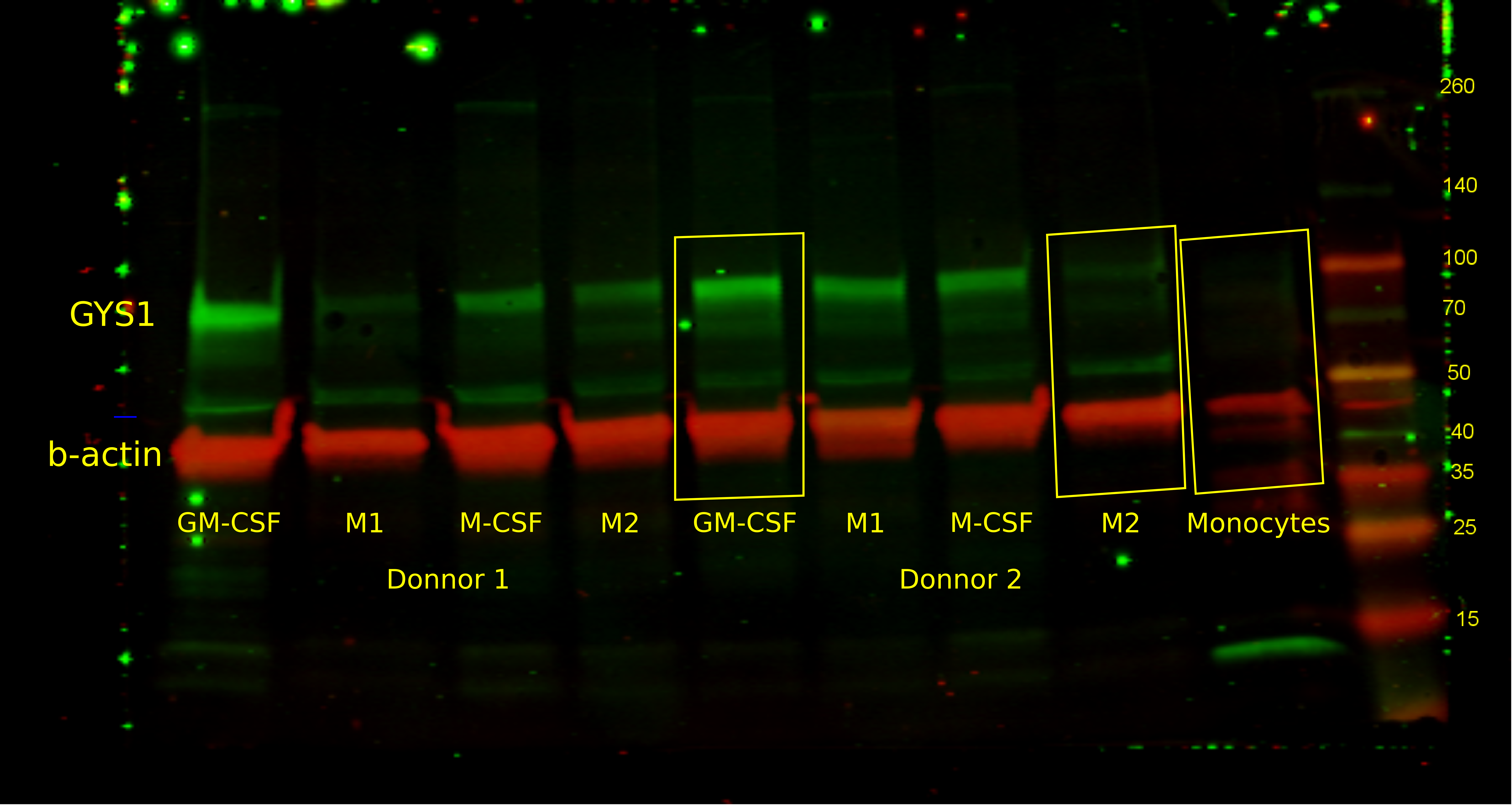

Supplement: Supplementary file 8 — Expanded View source data [file 44319_2024_278_MOESM8_ESM.zip › Expanded View/Expanded View Figure 1/EV1D/EV1_Western GYS1 b-actin.png]

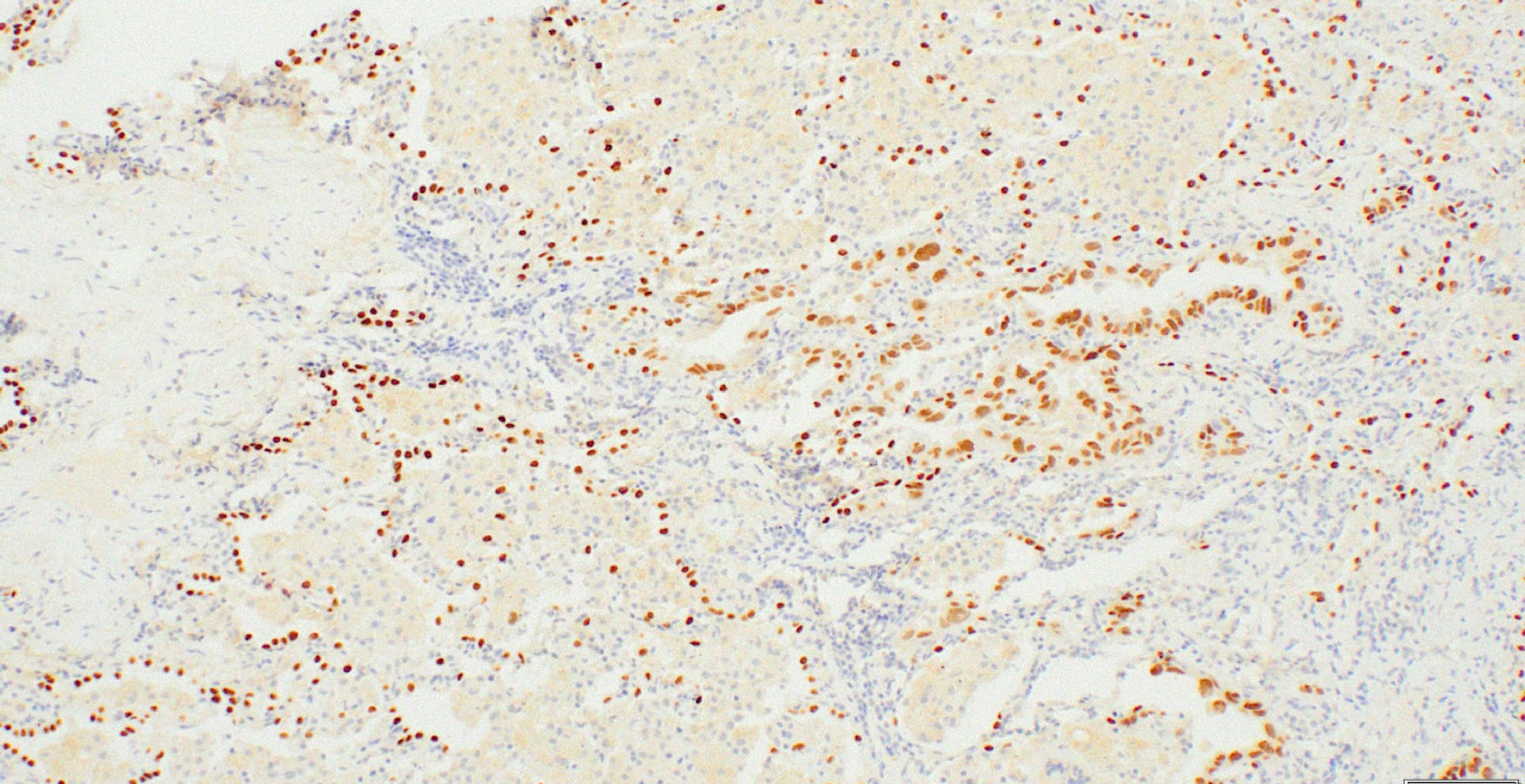

Supplement: Supplementary file 8 — Expanded View source data [file 44319_2024_278_MOESM8_ESM.zip › Expanded View/Expanded View Figure 4/EV4_IHC TTF-1 adenocarcinoma.png]

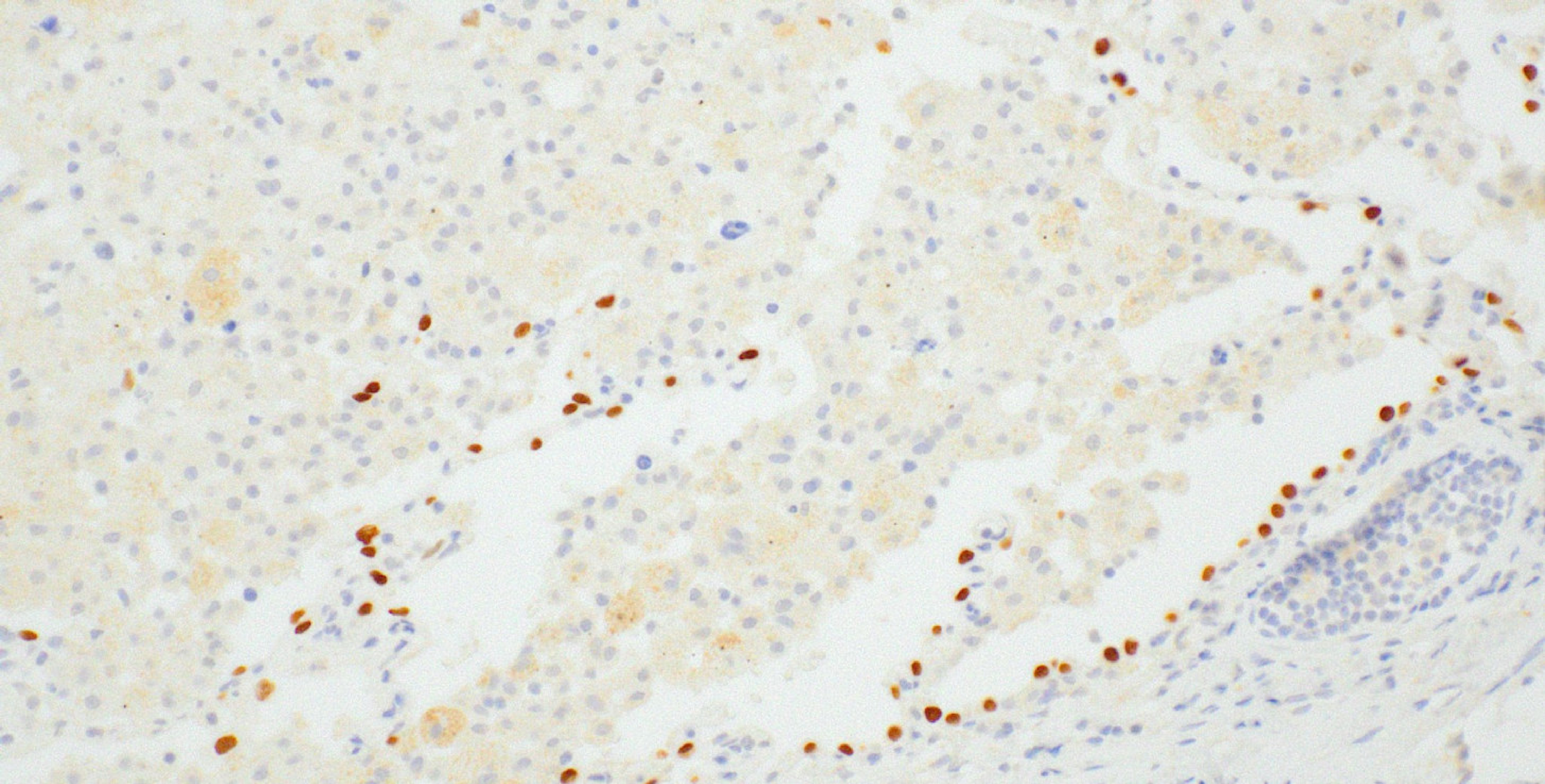

Supplement: Supplementary file 8 — Expanded View source data [file 44319_2024_278_MOESM8_ESM.zip › Expanded View/Expanded View Figure 4/EV4_IHC TTF-1 pneumocytes.png]
