# Supplementary material for: Glycogenesis and glyconeogenesis from glutamine, lactate and glycerol support human macrophage functions
Source: EMBO Rep. 2024 Oct 18;25(12):13. doi: 10.1038/s44319-024-00278-4 (PMC11624281; doi:10.1038/s44319-024-00278-4)
Supplement: Supplementary file 9 — Expanded View Figures [file 44319_2024_278_MOESM9_ESM.pdf]

Expanded View Figures

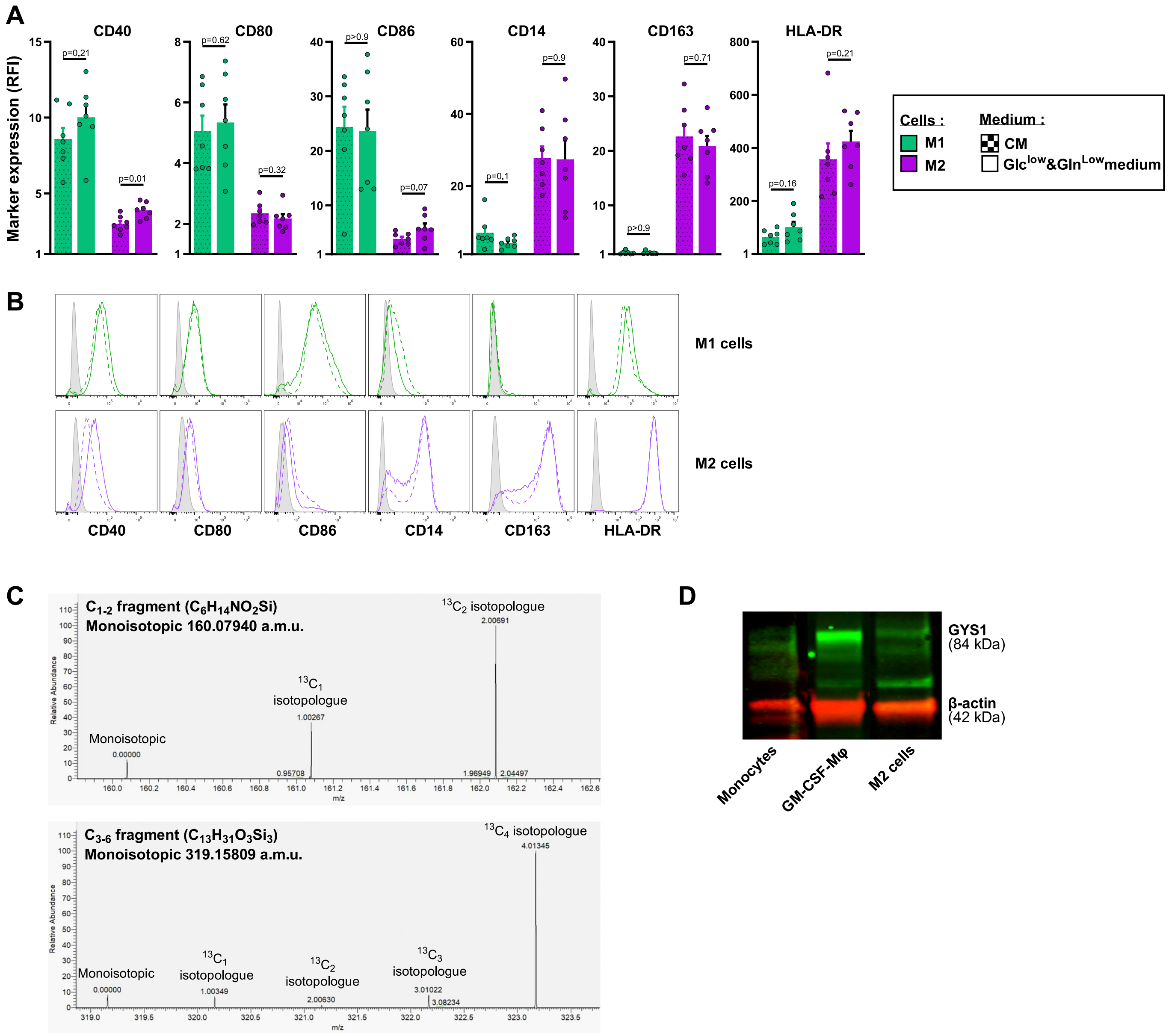

Figure EV1. GM-CSF or M-CSF plus IL-4 triggers human macrophage glycogenesis.

(**A**, **B**) Phenotypic characterization of M1 and M2 cells. M1 and M2 cells were generated either in conventional medium (CM) for 5 days or in CM for the first two days followed by 3 days culture in Glc^{low} Gln^{low} medium up to day 5. (**A**) The M1 versus M2 phenotype was analyzed by flow cytometry using BUV395-labeled anti-CD14, BV421-labeled anti-CD163, BV510-labeled anti-CD40, FITC-labeled anti-CD80, PE-cy7-labeled anti-CD86 and APC-labeled anti-HLA-DR mAbs (all from BD Biosciences). Results are expressed as relative fluorescence intensity (RFI) ($n = 7$). (**B**) Representative flow cytometry histograms. Gray histograms represent isotype controls, dashed lines and solid lines represent cells generated in CM and Glc^{low} Gln^{low} medium, respectively. (**C**) Isotopic patterns in Glc from glycogen show the prevalence of fully labeled isotopologues. Two typical fragments of 1-methoxy-penta-trimethylsilyl glucose (Glc 1MeOX, 5TMS) glucose are shown: (upper panel) isotopic pattern of the fragment at *m/z* 160.0794 a.m.u. corresponding to C-atom positions C1-C2, (lower panel) isotopic pattern of the fragment at m/z 319.15809 a.m.u. corresponding to C-atom positions C3 to C6. Peaks are labeled with the mass difference with respect to the monoisotopic (^{12}C) form. Note that due to the ^{13}C enrichment, Si isotopologues (29Si and 30Si at natural abundance) of the glucose derivative are small and thus not visible on this scale. (**D**) GYS1 expression was analyzed by western blotting in monocytes, day 5 GM-CSF-Mφ, and M2 cells; actin was used as loading control (representative of one out of 3). Values are represented as the mean ± SEM, each dot represents a different donor. Statistical significance was determined by two-tailed unpaired Welch *t* test (**A**). Source data are available online for this figure.

© The Author(s)

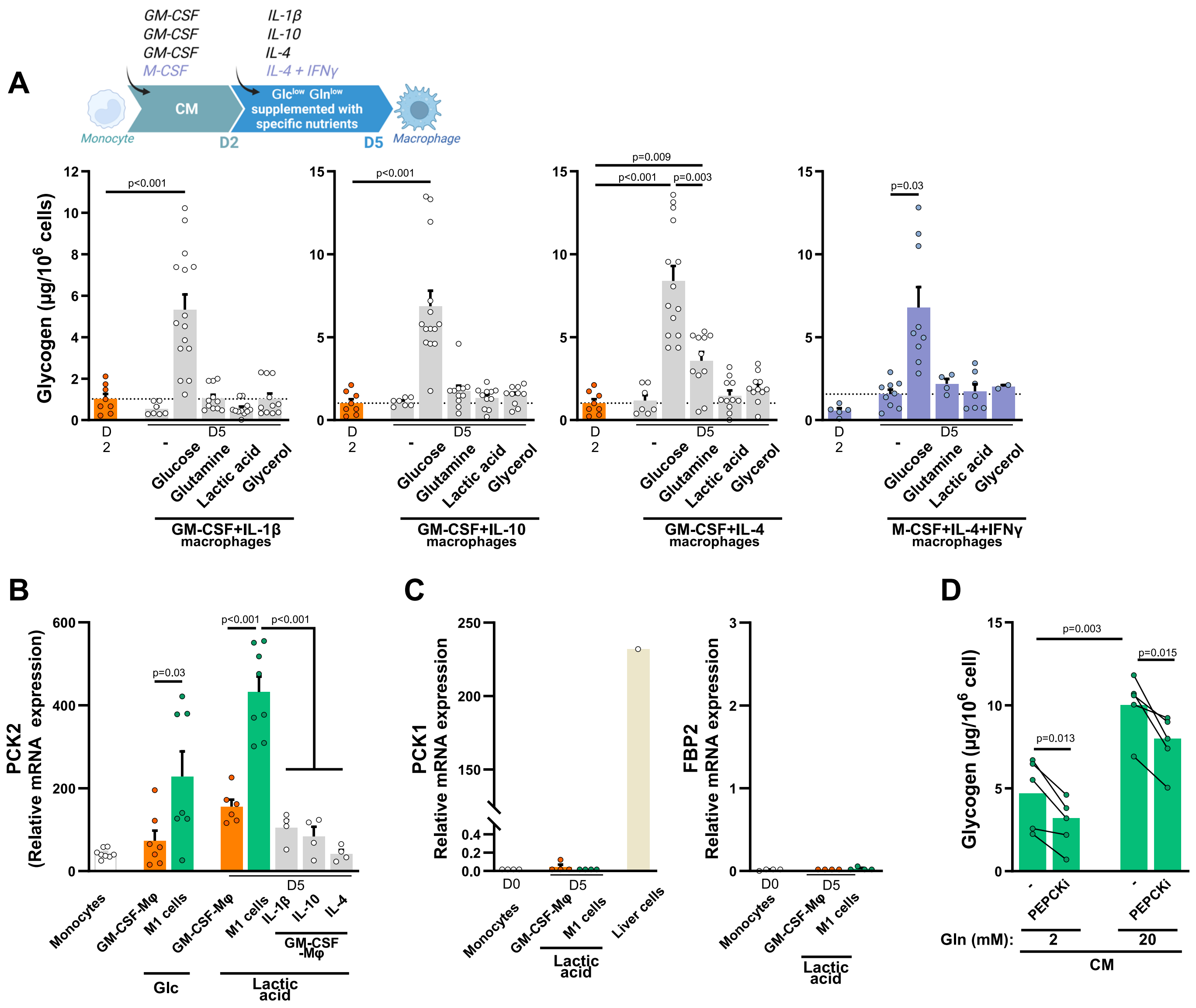

Figure EV2. The combination of GM-CSF plus IFNγ promotes human macrophage glyconeogenesis.

(**A–C**) Impact of cytokines on Mφ glyconeogenesis. GM-CSF-Mφ and M-CSF-Mφ were switched on day 2 to Glc^{low} Gln^{low} medium supplemented or not with the specific nutrients Glc, Gln, lactic acid or glycerol. The timelines summarize the experimental procedures for macrophage generation. (**A**) IL-1β, IL-10, IL-4, or IL-4 plus IFNγ were added or not during the differentiation and glycogen was quantified in day 5 Mφ ($n = 4$–15). (**B**) IL-1β, IL-10, IL-4 were added or not during the differentiation of GM-CSF-Mφ and PCK2 expression was determined by RT-qPCR ($n = 4$–8). (**C**) PCK1 and FBP2 expression was determined by RT-qPCR in monocytes, GM-CSF-Mφ and M1 cells. Liver cells were used as a positive control for PCK1 expression ($n = 4$). (**D**) M1 cells rely on glycogenesis and glyconeogenesis to synthesize glycogen. Glycogen content was determined in M1 cells cultured for 5 days in CM containing either 2 or 20 mM Gln, in the absence or presence of 10 µM PEPCKi ($n = 5$). Values are represented as the mean ± SEM, each dot represents a different donor. Statistical significance was determined by Welch's ANOVA test followed by Dunnett's multiple comparison post hoc test (**A**, **B**) or two-tailed unpaired Welch *t* test (**C**) or by paired *t* test (**D**). $^*P < 0.01$, $^{**}P < 0.005$, $^{***}P < 0.001$, $^{****}P < 0.0001$. Source data are available online for this figure.

 © The Author(s)

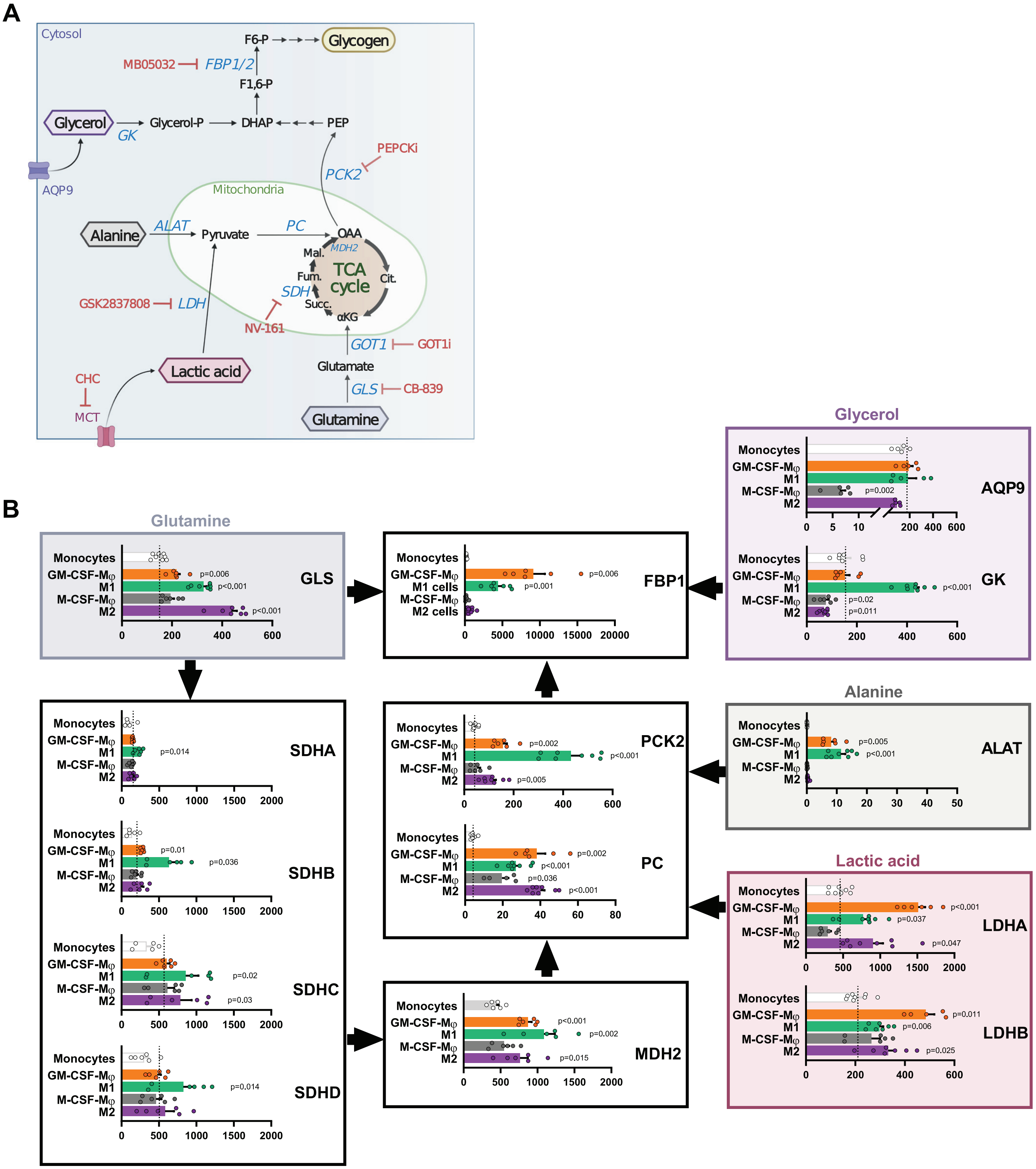

Figure EV3. Glyconeogenesis pathways.

(**A**) Main metabolites and enzymatic pathways involved in glyconeogenesis. Specific inhibitors used in this study are in red. (**B**) The expression of mRNA encoding enzymes involved in M1 glyconeogenesis (GLS, SDHA-D, FBP1, PCK2, PC, MDH2, AQP9, GK, ALAT and LDHA-B) was determined by RT-qPCR in monocytes and day 5 Mφ ($n = 6$–8). Values are represented as the mean ± SEM; each dot represents a different donor. Statistical significance was determined by Welch's ANOVA test followed by Dunnett's multiple comparison post hoc test. The *P* values represent the comparison between monocytes and macrophages. *$P < 0.01$, **$P < 0.005$, ***$P < 0.001$, ****$P < 0.0001$. Source data are available online for this figure.

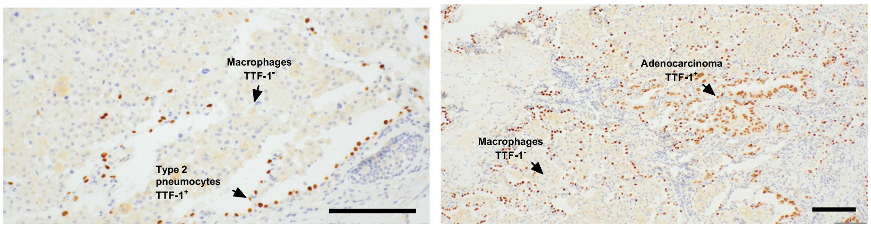

Figure EV4. Tumor-associated macrophages store glycogen and are capable of glyconeogenesis.

Immunohistochemical staining of TTF-1 in lung adenocarcinoma. Scale bar, 100 µm. Source data are available online for this figure.

 © The Author(s)

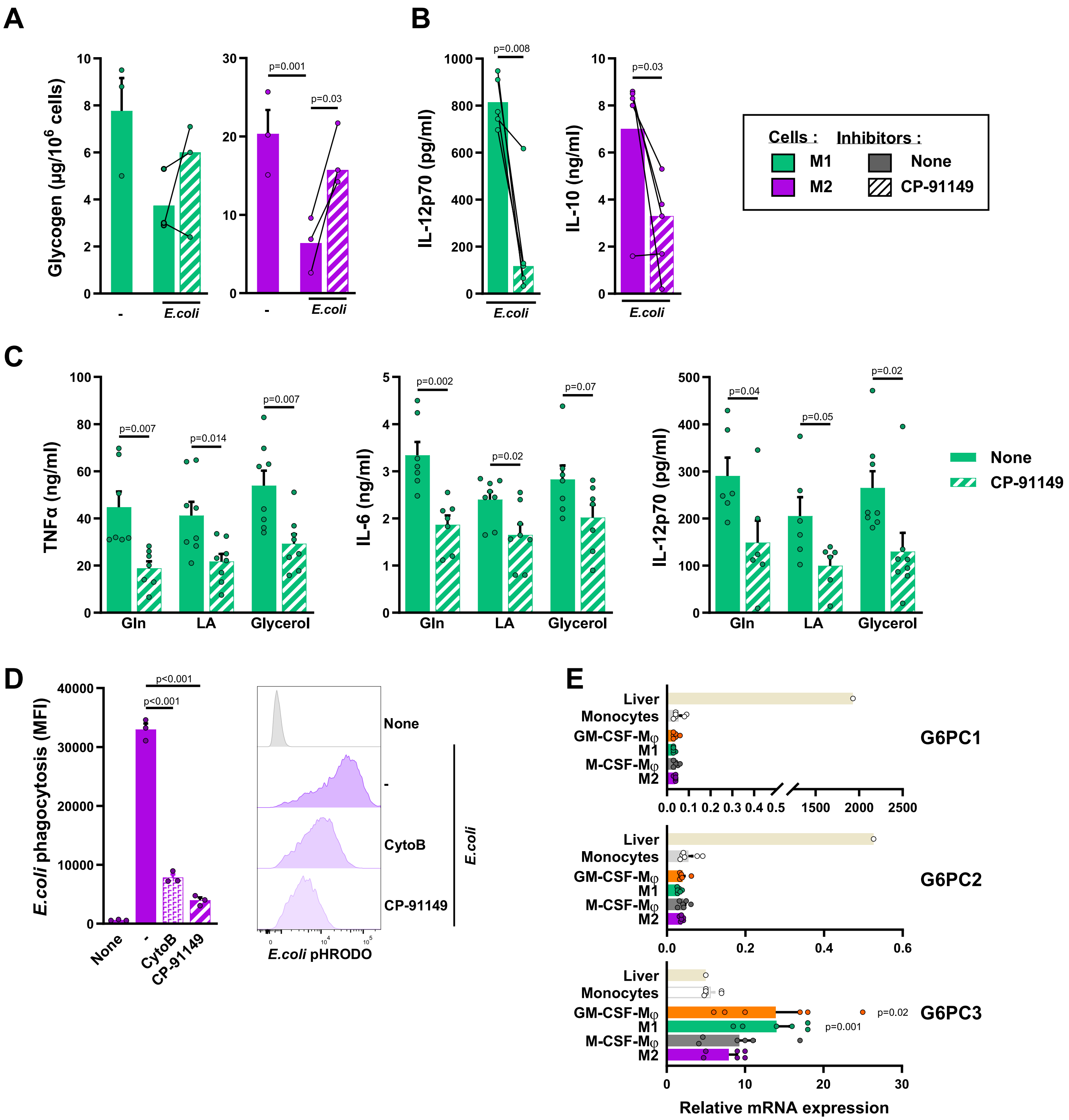

Figure EV5. Glycogen sustains M1 and M2 cell functions.

(**A, B**) Day 5 M1 and M2 cells (generated in conventional medium or CM) were treated or not for 15 min with 50 µM CP-91149 before stimulation with 100 ng/mL LPS or with *E. coli* at a multiplicity of infection of 10. (**A**) Glycogen was quantified after 24 h activation ($n = 3$). (**B**) Cytokines were quantified in the supernatants by ELISA after 24 h (IL-12p70) or 6 h (IL-10) stimulation ($n = 5$). (**C**) GM-CSF-Mφ were switched on day 2 to Glc^{low} Gln^{low} medium supplemented or not with Gln, lactic acid or glycerol and treated or not with CP-91149 15 min before LPS stimulation. TNFα and IL-6 (2 h), or IL-12p70 (24 h) were quantified by ELISA in stimulated day 5 M1 cells culture supernatants ($n = 6$–8). (**D**) M2 cells were pretreated with 50 µM CP-91149 or 10 µM cytochalasin B (CytoB) for 15 min before addition of 0.2 mg/mL pHrodo-conjugated *E. coli* BioParticles for 2 h at 37 °C. Fluorescence was determined by flow cytometry and expressed in MFI values ($n = 3$). (**E**) G6PC1-3 mRNA expression was determined by RT-qPCR in monocytes and day 5 Mφ ($n = 6$). Liver cells were used as a positive control. Values are represented as the mean ± SEM, each dot represents a different donor. Statistical significance was determined by paired *t* test (**A, B**) or by two-tailed unpaired Welch *t* test (**C, D**). $*P < 0.01$, $**P < 0.005$, $***P < 0.001$, $****P < 0.0001$. Source data are available online for this figure.

© The Author(s)
